# Evaluation of multiple displacement amplification for metagenomic analysis of low biomass samples

**DOI:** 10.1093/ismeco/ycae024

**Published:** 2024-02-12

**Authors:** Melody Cabrera Ospino, Katja Engel, Santiago Ruiz-Navas, W Jeffrey Binns, Andrew C Doxey, Josh D Neufeld

**Affiliations:** Department of Biology, University of Waterloo, Waterloo, Ontario N2L 3G1, Canada; Department of Biology, University of Waterloo, Waterloo, Ontario N2L 3G1, Canada; Department of Biology, University of Waterloo, Waterloo, Ontario N2L 3G1, Canada; Safety and Technical Research, Nuclear Waste Management Organization of Canada, Toronto, Ontario M4T 2S3, Canada; Department of Biology, University of Waterloo, Waterloo, Ontario N2L 3G1, Canada; Department of Biology, University of Waterloo, Waterloo, Ontario N2L 3G1, Canada

**Keywords:** multiple displacement amplification, low-biomass samples, metagenomic analysis, emulsion, primase, high-throughput sequencing, microbial community analysis

## Abstract

Combining multiple displacement amplification (MDA) with metagenomics enables the analysis of samples with extremely low DNA concentrations, making them suitable for high-throughput sequencing. Although amplification bias and nonspecific amplification have been reported from MDA-amplified samples, the impact of MDA on metagenomic datasets is not well understood. We compared three MDA methods (i.e. bulk MDA, emulsion MDA, and primase MDA) for metagenomic analysis of two DNA template concentrations (approx. 1 and 100 pg) derived from a microbial community standard “mock community” and two low biomass environmental samples (i.e. borehole fluid and groundwater). We assessed the impact of MDA on metagenome-based community composition, assembly quality, functional profiles, and binning. We found amplification bias against high GC content genomes but relatively low nonspecific amplification such as chimeras, artifacts, or contamination for all MDA methods. We observed MDA-associated representational bias for microbial community profiles, especially for low-input DNA and with the primase MDA method. Nevertheless, similar taxa were represented in MDA-amplified libraries to those of unamplified samples. The MDA libraries were highly fragmented, but similar functional profiles to the unamplified libraries were obtained for bulk MDA and emulsion MDA at higher DNA input and across these MDA libraries for the groundwater sample. Medium to low-quality bins were possible for the high input bulk MDA metagenomes for the most simple microbial communities, borehole fluid, and mock community. Although MDA-based amplification should be avoided, it can still reveal meaningful taxonomic and functional information from samples with extremely low DNA concentration where direct metagenomics is otherwise impossible.

## Background

Multiple displacement amplification (MDA) can increase DNA concentrations from low biomass samples prior to high-throughput sequencing. Such environments encompass deep subsurface locations, spacecraft, space stations, operation rooms, or samples like forensic specimen, single-cells, ancient samples, among others [1-6]. Although numerous studies have assessed the effectiveness and performance of MDA, particularly in amplifying viral metagenomes [7-9] and single-cell genomes [4, 10-14], few studies have explored its impact on microbial communities derived from mock communities or environmental samples [2, 15, 16]. Moreover, a subset of these studies have generated metagenomic data of amplified environmental samples, often focusing on taxonomic representation [2, 15, 17] and rarely examining the overall quality of assemblies, binning, or functional profiles.

MDA relies on the activity of the Phi29 polymerase, which amplifies DNA with high processivity and 3′-5′ proofreading activity [18], producing products with >10 kb average lengths and yields suitable for shotgun metagenomic sequencing [5, 19, 20]. Such MDA approaches have been used for amplifying DNA from isolated single cells by various methods [4, 12, 13, 21, 22] yet, despite these advantages, MDA can introduce coverage bias and artifacts because of differences in local template priming efficiencies and chimera formation [14, 23-25].

Several modifications to the MDA protocol have helped address limitations related to artifacts and bias. One approach is to reduce reaction volumes [14], by using microfluidic devices and droplet generators, limiting MDA reactions to a few cells or DNA molecules amplified to saturation [4, 13, 26]. For example, the use of microfluidics and emulsion protocols resulted in uniform amplification and high-coverage sequencing of genomes from *Escherichia coli* single cells [26]. Another modification involves the primase enzyme, such as “TruePrime” protocol. This method uses a DNA primase-polymerase to generate DNA primers from added dNTPs, followed by Phi29 polymerization and strand displacement. This modification improved the evenness of genome coverage and reduction in nonspecific amplification of a human cell line [27]. However, amplification bias to specific genome regions that vary among human cell line reaction replicates was still observed in another study [28]. Nevertheless, combining primase MDA with microfluidic-based metagenomic sequencing helped assemble multiple archaeal MAGs from an Obsidian Pool sample [29]. Although primase MDA have shown promise to reduce bias, to our knowledge, this method has yet to be evaluated in low-biomass environmental samples.

Previous research have compared the bulk MDA and primase MDA amplification bias on the microbial community after using amplicon sequencing, quantitative polymerase chain reaction (qPCR), and gel fingerprinting [3]. The present study tested different MDA-based amplification protocols (i.e. bulk MDA, emulsion MDA, and primase MDA) to analyze metagenomic data from amplified DNA obtained from mixed microbial communities. With replication and controls, we compared MDA protocols on two DNA template concentrations from a microbial community standard and two low biomass subsurface samples. Direct metagenomics without MDA amplification was used as an unamplified template comparison for the mock community and one environmental sample that had sufficient DNA for direct sequencing. Read mapping to known genomes of the community standard helped evaluate genome coverage, relative abundances, and chimera prevalence. We assessed the impact of these approaches on de novo assembly and binning and assessed how functional annotation profiles were influenced by each protocol. The results provide a foundation to support future studies using MDA-based amplification for metagenomic analysis of low biomass environmental samples.

## Materials and Methods

### Sample descriptions

We used the ZymoBIOMICS Microbial Community DNA Standard (D6305 Lot No. ZRC190812; Zymo Research, USA) as a well-characterized nucleic acid sample. This mock community features a diverse range of bacterial species with varying genome size and guanine–cytosine (GC) content (ranging from around 30% to 60%), suitable to effectively investigate the influence of these variables on MDA amplification performance. The mock community consisted of eight bacterial species, each represented with 12% DNA relative abundance by mass (i.e. *Pseudomonas aeruginosa*, *E. coli*, *Salmonella enterica*, *Lactobacillus fermentum*, *Enterococcus faecalis*, *Staphylococcus aureus*, *Listeria monocytogenes, Bacillus subtilis*) and two fungal species, each with 2% DNA abundance (i.e. *Saccharomyces cerevisiae*, *Cryptococcus neoformans*).

As representative environmental samples we selected a borehole fluid sample from the Grimsel Underground Research Laboratory in Switzerland [30] and a groundwater sample from ~40 m depth in Ontario, Canada. Both fluid samples were filtered through Sterivex-GV filters (Millipore, 0.22 μm, PVDF, USA), then placed in 50-ml plastic centrifuge tube and stored at −20°C. Although the specific volumes filtered by contractors for the borehole fluid and groundwater samples are unknown, they were anticipated to be no more than 1 L in total each. Sample DNA was extracted from Sterivex filters using the PowerSoil DNA Isolation kit (Qiagen, Germany), with bead beating to ensure rigorous cell lysis. The detailed DNA extraction procedure was reported in a previous study [30].

### Template quantification

Low biomass DNA quantities are defined as DNA concentrations that fall below the detection limits of traditional nucleic acid quantification methods, such as NanoDrop and Qubit, and insufficient for direct sequencing without prior amplification.The study used two picogram-ranged DNA quantities termed low and high levels. The mock community and borehole fluid samples were diluted with 10 mM Tris buffer (pH 8.5) to achieve DNA concentrations comparable to the groundwater sample for low-level analysis. For a high level, we select a lower dilution from the mock community and borehole fluid. To verify the genomic DNA concentrations resulting from our dilutions, we targeted 16S rRNA genes using qPCR with universal primers 341F and 926R [31]. The qPCR was performed in duplicate 15-μl reaction volumes consisting of 1× SsoAdvanced Universal SYBR Green Supermix (Bio-Rad, USA), 0.3 μM of each primer, 7.5 μg BSA, and 4 μl template on a CFX96 Real-Time PCR Detection System (Bio-Rad, USA). The qPCR conditions were 95°C for 3 min, followed by 40 cycles of 95°C for 15 s and 55°C for 30 s. In every run, we included no template controls (NTCs) using 10 mM Tris buffer used for qPCR dilutions, and “PCR water” (0.1 µm sterile filtered water treated with ultraviolet (UV) light) as the template. The number of bacterial 16S rRNA genes was calculated by comparing the amplification threshold to a standard curve prepared with purified PCR product that was amplified from a plasmid containing the 16S rRNA gene of *Thermus thermophilus* (positions 341–926). Standard curves showed >90% efficiency (except one, below 80%) and coefficients of determination (*R*^2^) >0.99.

Sample genomic DNA concentrations were estimated using 16S rRNA gene copy abundances by assuming an average bacterial chromosome size of 3.65 Mb [32], an average 16S rRNA gene copy number of 3.6 [33], and an average molecular weight of 650 g per mole of double-stranded DNA. With these estimates, the following equation was used to estimate genomic DNA concentrations from qPCR-based sample 16S rRNA gene copy numbers [34].


\begin{align*} \mathrm{DNA}\ \mathrm{concentration}\ \left[\frac{\mathrm{pg}}{\mathrm{\mu} \mathrm{l}}\right]=\left(16\mathrm{S}\ \mathrm{rRNA}\ \mathrm{gene}\ \mathrm{copies}\right)\\\left(\frac{\mathrm{genome}\ \mathrm{size}\ \left[\mathrm{bp}\right]}{16\mathrm{S}\ \mathrm{rRNA}\ \mathrm{gene}\ \mathrm{copies}\ \mathrm{per}\ \mathrm{genome}}\right) \end{align*}

\begin{align*} \times \left(\frac{650\ \mathrm{g}\ \mathrm{DNA}}{1\ \mathrm{mol}\ \mathrm{bp}\ \mathrm{DNA}}\right)\left(\frac{1\ \mathrm{mol}\ \mathrm{bp}}{6.02\ \mathrm{X}\ {10}^{23}\ \mathrm{bp}\Big)}\right)\left(\frac{10^{12}\ \mathrm{pg}}{1\ \mathrm{g}}\right)\\\left(\frac{1}{\mathrm{volume}\ \mathrm{of}\ \mathrm{template}\ \left[\mathrm{\mu} \mathrm{l}\right]}\right) \end{align*}

### Multiple displacement amplification

We tested three MDA protocols in this study: bulk MDA, primase MDA, and emulsion MDA. Furthermore, we used two DNA input concentrations, referred to as “high” and “low” (Table 1), although available DNA from the groundwater sample was only sufficient for the low DNA input treatment. Two negative controls were included that contained PCR water and 10 mM Tris buffer instead of DNA template. Each sample was amplified in duplicate for each protocol. All MDA reactions were prepared in a workstation with ISO 5 HEPA-filtered air (Air Clean Systems, Canada). Surfaces and pipettes were cleaned with sodium hypochlorite and 80% ethanol followed by UV irradiation for 15 min. Tubes, PCR water, and tube racks were UV-treated for 30 min with 302 nm light on a transilluminator (Protein Simple, USA). The reagents were thawed and maintained on ice throughout the experimental setup. The DNA yields of MDA treatments were determined using the Qubit dsDNA High Sensitivity assay kit (Invitrogen, USA) and measured the fluorescence intensity on a plate reader (FilterMax F5 MultiMode, Molecular Devices, USA) at excitation and emission wavelengths of 485 and 525 nm, respectively.

**Table 1:** DNA amplification yield after bulk MDA, emulsion MDA, and primase MDA.

Sample	16S rRNA gene copies	DNA amount (pg)	Replicate name	Bulk MDA yield (μg)	Emulsion MDA yield (μg)	Primase MDA yield (μg)
Mock community	202 365	110	High-1	1.3	0.7	1
			High-2	2.2	1.1	1.2
	1632	0.8	Low-1	2.5	1.7	1.7
			Low-2	2	1.9	1.2
Borehole fluid	152 060	83	High-1	2.2	1.5	1.3
			High-2	2.3	1	1.5
	1561	0.8	Low-1	2.7	1.3	0.1
			Low-2	2.4	1.6	0.04
Groundwater	1030	0.6	Low-1	2.2	1.5	0.7
			Low-2	2.2	1.1	0.6
PCR water	N/A	N/A	N/A	0.1	<LOD	<LOD
PCR Tris buffer	N/A	N/A	N/A	0.1	<LOD	<LOD

N/A = not applicable

<LOD = below limit of detection of the Qubit dsDNA High Sensitivity assay

For bulk MDA (Illustra Single Cell GenomiPhi DNA Amplification Kit, GE Healthcare, UK), 2 μl of templates were combined with 1 μl of the prepared GenomiPhi lysis buffer and incubated for 10 min at room temperature. Although the manufacturer’s protocol recommends only 1 μl of template, the template concentration we used does not exceed the amount that inhibits Phi29 DNA polymerase (>1 ng). After 10 min incubation, 1 μl of single-cell GenomiPhi neutralization buffer was added to stop the DNA denaturation reaction. Amplification mix was prepared beforehand and left on ice until needed. To the denatured mixture, 16 μl of this amplification mix was combined by pipetting, centrifuged briefly, and the 20 μl reaction was incubated at 30°C for 4 h. The amplification reaction was inactivated at 65°C for 10 minutes, cooled to 4°C, and the product was stored at −20°C.

For emulsion MDA, the same kit as for bulk MDA was used and a 20 μl MDA reaction mixture was prepared as described above. Immediately after the MDA reaction was mixed, an emulsion was created using a vortex following the protocol described elsewhere [26]. Briefly, emulsion MDA was performed by adding 20 μl of a 1% (w/w) Perfluoropolyether-polyethylene glycol-perfluoropolyether (PFPE-PEG-PFPE) amphiphilic block copolymer surfactant (catalogue no. 008, RAN Biotechnologies, Inc., USA), dissolved in HFE-7500 fluorinated oil (RAN Biotechnologies, Inc., USA), to the MDA reaction mixture. The mixture was then vortexed for 10 s to create droplets and incubated at 30°C for 4 h. The amplification reaction was inactivated at 65°C for 10 min. To the amplified reaction, we added 10 μl of 1H,1H,2H,2H-perfluoro-1-octanol to disperse droplets. The amplified product aqueous phase was retrieved after centrifugation and stored at −20°C.

For primase MDA (TruePrime Single-cell WGA kit, Sygnis, Germany), 2 μl of samples were combined with 2 μl of the denaturing buffer, mixed by pipetting, and incubated for 3 min at room temperature. Subsequently, 2 μl of neutralization buffer was added to stop the reaction. Next, 44 μl of amplification mix was added and incubated at 30°C for 4 h. The amplification reaction was inactivated at 65°C for 10 min, cooled to 4°C, then stored at −20°C. All MDA reactions were purified using ethanol precipitation as described in Illustra Single Cell GenomiPhi DNA Amplification Kit. The DNA was suspended in 10 mM Tris buffer and stored at −20°C.

### Shotgun sequencing

Metagenomic libraries were generated for each duplicate amplification treatment, unamplified templates, and two negative controls, using the NEBNext Ultra II DNA Library Prep Kit for Illumina (New England BioLabs, Ipswich, MA). The DNA input for library preparation ranged from ~210 ng to ~8 ng based on the DNA concentration of the sample. Library fragment size was determined with the Genomic DNA ScreenTape assay (Agilent) with an average insert size of 650 to 710 bp. After library preparation, the concentration for pooling was adjusted to a fixed number of clusters: 4 000 000 for mock community and borehole fluid, aiming for an approximate 15× sequencing depth based on the microbial structure. Groundwater was assigned 32 000 000 clusters due to its complex microbial community revealed by prior 16S rRNA amplicon sequencing (Unpublished data). Libraries were sequenced on two lanes of a HiSeq 2500 System (Illumina) in rapid run mode with 2× 250 base reads. The library preparation, normalization according to cluster counts and pooling, quality checking, and sequencing were performed at the McMaster Genome Facility (McMaster University, Hamilton ON, Canada).

### Bioinformatics

Raw sequences (FASTQ files) underwent quality checks and trimming using the metagenomic data processing pipeline ATLAS version 2.0.6 (Supplementary Data S1) [35]. Libraries exhibited similar sequence abundance ranges, except for one normalized to match its replicate's count (Supplementary Table S1). Taxonomic profiling of quality-checked reads was performed using the MetAnnotate pipeline [36] with the default parameter *E*-value (0.001) and sequence identity (50%) thresholds using hidden Markov model (HMM) single-copy gene markers profiles for *rpoB* and *gyrB* downloaded from FunGene. The reads were translated to amino acid sequences using FragGeneScan-Plus [37], and subsequently used to identify homologs in the National Center for Biotechnology Information (NCBI) Refseq database (release 204). The *rpoB* gene profile data generated by MetAnnotate were used to compute Bray–Curtis dissimilarity matrices, which were then used for corresponding principal coordinates analysis (PCoA) and permutational multivariate analysis of variance (PERMANOVA) in R using the package phyloseq [38] and vegan [39]. The PCoA and PERMANOVA analysis were made to visualize and evaluate the similarities of communities across different MDA methods and DNA input levels within each sample.

For the mock community metagenomes, we processed quality-filtered paired-end reads for each library by simultaneously mapping them to the eight bacterial reference genomes using BBSplit (BBtools package, http://sourceforge.net/projects/bbtools/). We retained only the first best match for ambiguous reads at a ≥ 76% identity threshold. This mapping split the reads in distinct FASTQ files for each reference genome and one for unmapped reads for every library. The resulting output was used to determine the read mapping proportion to each reference genome across libraries. Additionally, we assigned taxonomy to the unmapped reads using Kaiju v. 1.6.2 [40] with the NCBI RefSeq database to identify its sources (e.g., DNA contamination, sequencing error, or low-quality reads). We also employed a custom script to detect inverted and direct chimeric reads.

The FASTQ files were mapped back to the corresponding reference genome using BBMap (BBtools) with a 95% identity threshold to capture unique reads closely aligned with the reference genomes. The mapping results were saved as SAM/BAM and used for coverage profiles analysis, Lorenz curve plotting, and coverage standard deviation calculation.

Coverage profiles were generated for each reference genome across library using BEDTools (v. 2.29.0) coverage -hist. Pearson correlations between these profiles were calculated and visualize in two dimensions using the R package htSeqTools v. 1.30 [41]. The package provided an *R*^2^ coefficient, analog to the percentage of explained variability in a PCA analysis, which helped assess how well the plot's data points represented the original distances between the data. To evaluate coverage uniformity, we computed the coverage standard deviation using the function ssdCoverage of the htSeqTools package. Additionally, we generated Lorenz curve of coverage for each reference genome using the R package ineq v 0.2–13 [42], illustrating the cumulative fractions of the genome relative to the cumulative fraction of mapped reads. To evaluate distribution of read coverage, we also plotted the cumulative fraction of the genome covered (breadth of coverage) by a certain minimum depth. A detailed explanation of Kaiju, htSeqTool, and custom chimera detection script is provided in the Supplementary Data S2.

To explore GC bias, we aligned unique paired-end reads to four reference genomes, chosen to serve as representative samples from the mock community with different average GC content ranging from 30% to 66% (i.e. *S. aureus* 32.9%, *B. subtilis* 43.9%, *S. enterica* 52.2%, and *P. aeruginosa* 66.2%) using BBMap. The BAM file resulting from the alignment was used to compute the GC bias using Benjamini’s method [43], with help from the computeGCBias function of the deepTools package [44], using a window size equal to the read fragment length of 250 nt. ComputeGCBias function counts the number of reads per GC fraction and compares them to the expected GC profile, calculated by counting the number of DNA fragments per GC fraction in a reference genome.

Using ATLAS 2.0.6, all quality-controlled reads were assembled and binned without co-assembly. For de novo assemblies, we used MEGAHIT [45] and SPAdes [46] to determine the optimal assembler for MDA-amplified environmental samples. Before using SPAdes, the *k-*mer coverage was normalized to 10× and 40× with BBNorm from the BBTools program available in the ATLAS pipeline. Assembly size statistics were generated with ATLAS, and genes were predicted for contigs with Prodigal [47] using the default option mode –p: meta. The quality for each of the metagenome assemblies in the mock community was assessed using MetaQUAST [48] by mapping the contigs back to the reference genomes and comparing total genome coverage. MetaQUAST also reports the number of misassembled contigs based on the structural and sequence disagreements between contigs and reference genomes. Metagenomics binning for each assembly was generated by implementing CONCOCT [49], MetaBAT 2 [50], and MaxBin [51] methods, following dereplication and refinement by DASTool [52] with a minimum score of 0.3. Bin completeness and contamination were assessed using CheckM [53]. Detailed parameter options executed in the ATLAS pipeline for quality checking, trimming, assembly, and binning are detailed in Supplementary Data S1. Bin quality was based on completion and contamination as described elsewhere [54]: “high-quality” was defined as >90% completion and <5% contamination, “medium-quality” as ≥50% completion and <10% contamination, and “low-quality” as <50% completion and <10% contamination. Predicted genes from each assembly obtained from SPAdes 40× coverage normalization were annotated with a given Kyoto Encyclopedia of Genes and Genomes (KEGG) orthology (KO) using KofamKOALA [55] and parsed through the KEGG-Decoder to determinate the completeness of metabolic pathways [56].

For environmental sample libraries, read taxonomy profiling was performed using MetAnnotate as described above. Library quality control, trimming, assembly, gene prediction, and binning were conducted using the mock community workflow with the following modifications in ATLAS. Borehole fluid libraries were assembled with SPAdes without *k-*mer normalization, given the lower complexity of the microbial community. The groundwater libraries were assembled using SPAdes after k-mer normalization to a 40× sequencing depth. This adjustment was necessary to address the elevated coverage imbalance, particularly pronounced in this highly complex microbial community. Gene annotation was made using KofamKOALA. Completeness of metabolic pathways was determined using KEGG-Decoder.

The taxonomic distribution of reads from the raw reads of the no-template control from the MDA amplification methods (PCR water and PCR Tris buffer) libraries was analyzed using the NCBI SRA Taxonomy Analysis Tool. This online tool maps sequencing read matches to a precomputed k-mer dictionary of the RefSeq genomic database. The tool reports the relative abundance composition proportional to the sequence abundance of the genome of the reads organized in a taxonomic hierarchy [57].

### Statistical analyses

All statistical analyses and visualizations were performed with R version 4.0.5. The data size and the number of pathways detected in each library’s assembly functional profile were used as input for does Analysis of Variance (ANOVA) comparing the MDA method and input level.

## Results

### Amplification yields

Three MDA protocols (bulk MDA, primase MDA, and emulsion MDA) were tested using high (~100 pg) and low (~1 pg) genomic DNA concentrations determined through estimations of the 16S rRNA gene copy numbers. High and low DNA input quantities were obtained for the mock community and borehole fluid. Only the low input quantity was possible for the groundwater sample (Table 1). All protocols amplified DNA templates by over 1000-fold, with the bulk MDA protocol producing the highest yields (Table 1). Primase MDA had lower yields than emulsion MDA for low DNA inputs in borehole fluid and groundwater samples, even though yields were similar for the mock community. Negative control amplifications were below the limit of detection for primase and emulsion MDA, but bulk MDA generated amplification products with at least one order of magnitude less DNA yield than for all sample amplifications (Table 1).

### Dataset size

The groundwater libraries exhibited the highest average data size of 9.8 Gb (Supplementary Table S1) due to a larger number of assigned clusters during sequencing. In contrast, the borehole fluid and mock community libraries had smaller sequence dataset sizes of 1.2 Gb and 1.6 Gb, respectively, showing similar magnitudes because they were sequenced using the same number of clusters. Excluding the groundwater sample, no significant differences were detected in the sizes of quality checked and trimmed data between the libraries from the negative controls, unamplified samples, and samples amplified with all MDA protocols (*p* = .9).

### Taxonomic profiles

**Figure 1:**
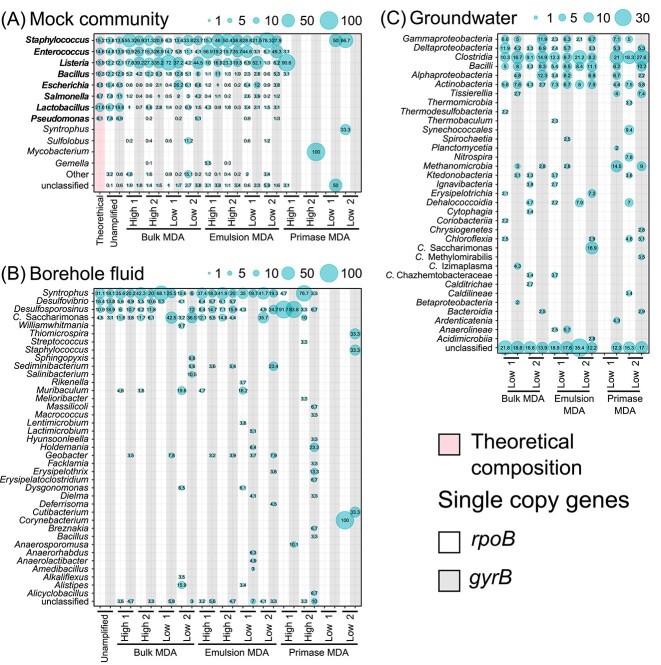
Bacterial taxonomic profiles for the mock community (A), borehole fluid (B), and groundwater (C) libraries amplified with bulk, emulsion, and primase MDA; high and low DNA input amounts were tested for the mock community and borehole fluid, but low DNA inputs were only possible for groundwater; an unamplified control library was included for the mock community and the borehole fluid; the theoretical composition of the mock community is shown first; genera at or above 0.1% (A), ≥3% (B), or class at ≥2% (C) relative abundance are shown for *rpoB* and *gyrB* HMMs hits, the category “other” in panel A corresponds to genus below 3% gene abundance in all libraries; gene abundance is indicated by the size of the circles.

We compared the consistency of taxonomic profiles derived from the *rpoB* and *gyrB* genes detection and phylogenetic affiliation in the quality-checked reads of the MDA-amplified and unamplified libraries for the mock community and borehole fluid (Fig. 1A and B). The groundwater sample had extremely low DNA concentration for direct sequencing; thus, only the MDA-amplified libraries were compared (Fig. 1C). There were no *rpoB* or *gyrB* genes identified in the no-template control libraries amplified using the bulk MDA method (i.e. water only or Tris buffer) and subsequent assembly attempts failed (data not shown).

Taxonomic identification of the bulk MDA amplified NTC libraries revealed that most raw reads from the NTC Tris buffer sample (99.8%) could be classified (Supplementary Fig. S1). Among these, 68% were assigned to the *Eukaryota* domain, with 30.2% of those belonging to *Homo sapiens*. Bacteria were identified in 23.3% of the reads, primarily from the *Terrabacteria* group (21.1%), with *Actinobacteria* (11.4%) being the dominant class, specifically from the *Propionibacteriaceae* family (8.8%). A minimal proportion of reads (<0.1%) were classified as members of the mock community genomes (Supplementary Fig. S1).

For the NTC PCR water library, 86.8% of the reads were taxonomically classified, with the majority (73.4%) assigned to the *Eukaryota* domain, specifically to the *Homininae* subfamily (62.1%) (Supplementary Fig. S1). Less than 1.5% of reads were classified as bacteria, and <0.1% were identified at lower taxonomic resolutions, including mock community members and other bacterial taxa (Supplementary Fig. S1).

For the mock community, we also included the expected profile provided by the manufacturer, with all eight bacterial representatives having a similar relative abundance to that of the unamplified library (Fig. 1A). The taxonomic profile in MDA-amplified libraries differed from the expected distribution (Fig. 1A). *Pseudomonas* reads were infrequently identified in bulk MDA and emulsion MDA libraries (below 1%), whereas the remaining reference taxa were consistently detected, but at different relative abundances. The primase MDA libraries mainly identified *S. aureus*, with no *rpoB* or *gyrB* genes detected in some replicates (Fig. 1A). *Mycobacterium* and *Syntrophus* were detected in one replicate of the primase MDA high and low libraries, but these were likely contaminants coming from other processed samples. Based on the PCoA plot, bulk MDA and emulsion MDA libraries community composition were closer to the unamplified library than for primase MDA, and no grouping was observed based on the DNA input level (Supplementary Fig. S2A). There was no evidence that the MDA methods (*p* = .3) nor DNA input (*p* = .5) influenced the community composition profiles.

For the borehole fluid sample, the taxonomic profile of the unamplified library detected the same abundant taxa (*Syntrophus*, *Desulfovibrio, Desulfosporosinus*, and *Candidatus* Saccharimonas) in both *rpoB* and *gyrB* genes of the high DNA input libraries of bulk MDA and emulsion MDA (Fig. 1B). Primase MDA failed to generate a taxonomic profile visually similar to the unamplified library. However, the high DNA input libraries could detect a few of the abundant taxa in increased relative abundances, *Syntrophus* and *Desulfosporosinus* (Fig. 1B). *Corynebacterium*, *Thiomicrospira*, and *Holdemania* were observed in the primase MDA low DNA input libraries and in the unamplified library at lower abundance (<1%). Other taxa identified below 1% abundance of the unamplified library were the archaea *Halobacteria* and *Methanomicrobia*. The PCoA analysis showed that communities in high DNA input bulk MDA and emulsion MDA libraries grouped together with the unamplified library, suggesting community composition overlap among these samples (Supplementary Fig. S2B). The ordination also suggested that communities of one replicate in low DNA input bulk and emulsion MDA and one high DNA input primase MDA libraries were closer to the unamplified library. Other primase MDA libraries showed a clear trend to diverge further from the corresponding unamplified library. The PERMANOVA test revealed strong evidence that MDA methods affected the difference in community composition (*p* = .03) but not DNA input (*p* = .46).

The groundwater sample had limited DNA concentration and only the low DNA input could be tested for all three MDA protocols. Taxonomic profiling identified six class-level taxa present in both *rpoB* and *gyrB* profiles at ≥2% relative abundance for all libraries, except for one replicate of primase MDA (low-1) that failed to produce any *rpoB* gene reads (Fig. 1C). The most abundant taxa were *Clostridia*, *Bacilli*, and *Actinobacteria*, followed by *Gamma-*, *Delta-*, and *Alphaproteobacteria.* Sequences from archaea were detected, with *Methanomicrobia* being the most abundant class (2.8%) using the *gyrB* gene. Other archaeal classes, such as *Halobacteria* and *Thermoplasmata*, were also present, albeit at lower abundances (<1%). In the case of the *rpoB* gene, these classes were identified along with others from the phyla *Euryarchaeota*, *Crenarchaeota*, and *Thaumarchaeota*, with an abundance below 1%. Ordination analysis showed that the bulk MDA libraries and one replicate of the emulsion MDA were closer suggesting more similar communities (Supplementary Fig.S2C). PERMANOVA test showed that MDA method did not significantly influence the observed differences in the community composition.

### Amplification bias and nonspecific amplifications

**Figure 2:**
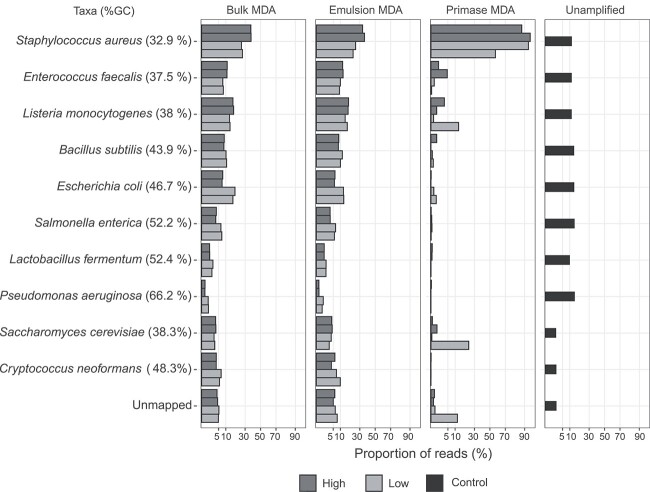
Proportion of quality-checked reads mapped to the reference genome for each mock community member; high (dark gray) and low DNA input (light gray) libraries are shown for each MDA amplification method as well as the unamplified control library; the reference genomes are sorted by GC content (indicated in brackets) from top to bottom.

We assessed each MDA method’s amplification bias and read distributions using a defined mock community sample. The unamplified library showed an expected read distribution, with proportions similar to the reported genomic DNA proportion for each bacterial or fungal member (12% or 2%, respectively; Fig. 2). However, this was not observed for bulk MDA and emulsion MDA. The proportion of mapped reads decreased for DNA genomes with higher GC content. Most reads of the Primase MDA high and low DNA input libraries mapped to *S. aureus* (50%–90%). Thus, other mock community members were not sufficiently covered, as seen in the community profiles discussed previously (Fig. 1A).

**Figure 3:**
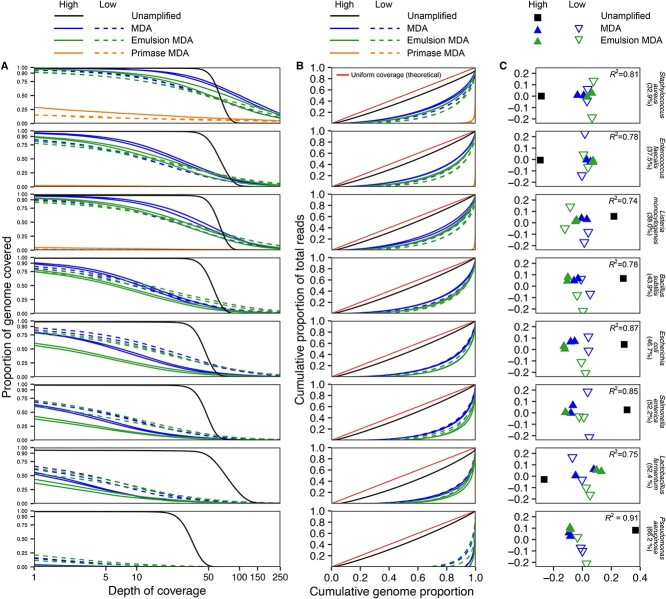
Coverage profiles for reference genomes in the unamplified and MDA-amplified mock community libraries, distinguished by different DNA input amounts (i.e. High and Low); the reference genomes were sorted based on their GC content, which increases from the top to the bottom; (A) the relationship between the genome’s coverage depth and breadth; (B) Lorenz curves illustrate coverage uniformity across the genome (with an ideal uniform coverage curve depicted for comparison); (C) ordinations that compare coverage profiles across the libraries using nonmetric multidimensional scaling (NMDS) based on Euclidean distances of log-transformed coverage profiles.

The unamplified library showed over 95% genome breadth of coverage (i.e. the proportion of the genome covered by reads) and over 15× sequencing depth (i.e. the average number of reads aligned to a given position in the genome) for all bacterial members of the mock community (Fig. 3A). However, bulk MDA and emulsion MDA resulted in higher breadth of coverage and mapping depth for low GC content and small-sized genomes (i.e. *S. aureus*, *E. faecalis*, *L. monocytogenes*, and *B. subtilis*), with >50% of these genomes covered with at least 10× depth in the high DNA input libraries (Fig. 3A). The coverage decreased with increasing GC content genome in both bulk and emulsion MDA methods. Although greater than 90% of the low GC content genome of *S. aureus* was covered by both MDA methods, <25% of the high GC genome of *P. aeruginosa* was covered. Primase MDA libraries had very poor coverage of most mock community genomes, showing only 15%–30% coverage of the low GC content genome *S. aureus* with over 90× sequencing depth (Fig. 3A). We assessed amplification uniformity using coverage Lorenz curves (Fig. 1B). The curve angles closer to the 45-degree line indicate high uniformity, and further away suggests uneven read distribution across the genome. The unamplified library, which worked as a control, was closely observed to the 45-degree line. Bulk and emulsion MDA exhibited increasing uniformity bias with larger genomes and higher GC content. Conversely, Primase MDA showed the highest coverage bias across all reference genomes (Fig. 3B).

Coverage uniformity was also assessed using coverage standard deviation, henceforth referred to as ssdCoverage, measuring how evenly each genome within the library was amplified (Supplementary Fig. S3). Low ssdCoverage indicates even coverage, whereas high values suggest uneven amplification. The unamplified library generally had lower ssdCoverage for most genomes, except *L. fermentum*. In contrast, *S. aureus*, often highly covered in all bulk and emulsion MDA libraries, has the highest ssdCoverage suggesting an amplification imbalance. In contrast, *P. aeruginosa* had lower ssdCoverage, possibly due to fewer mapped reads in all libraries. Primase MDA was excluded due to insufficient mapping to most reference genomes for ssdCoverage computation (Supplementary Fig. S3).

Read distribution, coverage completeness, and uniformity results showed that reads nearly completely covered lower GC-content reference genomes. However, these genomes were also prone to the potential over and underamplification of some regions. Examining a segment of the *S. aureus* genome, we observed alternating regions of low and high coverage depth for all MDA libraries. Primase MDA showed particularly distinct patterns, with some parts having no coverage (0× depth), followed by others with exceptionally high coverage (over 100×) on the same fragment (Supplementary Fig. S4).

The GC bias was further analyzed through read recruitment to genomes from the mock community libraries (Supplementary Fig. S5). The results showed that all MDA amplified libraries demonstrated low coverage of GC fraction over 45% or higher; thus, the *P. aeruginosa* genome (66.2% GC) was underrepresented. In contrast, the unamplified library showed good amplification across GC fractions from 20% to 70% (Supplementary Fig. S5). The log coverage profiles ordinations revealed that the distances between the high input DNA bulk MDA and emulsion MDA for almost all of the reference genomes and their replicates were shorter than to the corresponding lower input libraries or unamplified libraries, suggesting a similarity in coverage patterns (Fig. 3C).

Between 1% and 8% of unmapped reads were found in most of the libraries, with the exception of one replicate in primase MDA up to 12% (Fig. 2). Of these unmapped reads, <0.07% were classified as chimeras, with the majority identified as inverted (Supplementary Fig. S6A). The segments of the chimera reads spanned at least 20 bp, reaching up to 5000 bp apart. Most chimeras were ~500 bp apart on the same DNA fragment (Supplementary Fig. S6B). The segments from these chimeras originated from the same DNA sequences. Among the total of unmapped reads that did not exceed 5% in bulk MDA, 8% in emulsion MDA, and 12% in one replicate of the primase MDA, <1.3% were affiliated to members of the mock community, others did not exceed 0.6% for other known taxa, and the majority could not be classified taxonomically (Supplementary Fig. S7).

### Read assembly and binning

**Figure 4:**
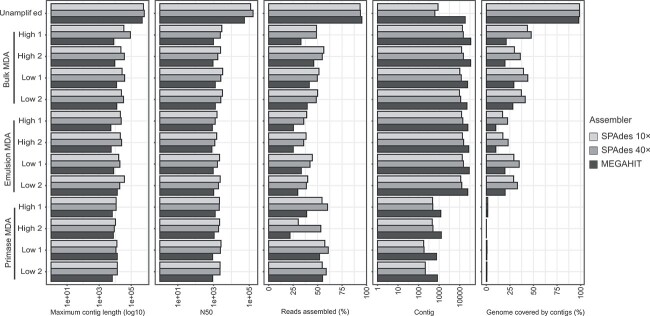
Assembly statistics for the unamplified and MDA libraries from the mock community; high and low-DNA input samples were assembled with SPAdes using 10× or 40× *k-*mer coverage normalization and MEGAHIT.

The mock community metagenomes were assembled using SPAdes (10× and 40× *k-*mer depth normalization) and MEGAHIT to evaluate the performance of different assembly algorithms in handling metagenomic data amplified by MDA. The unamplified libraries generally yielded longer contigs, higher N50 values, and a greater proportion of assembled reads for all assemblers. However, when using MEGAHIT, the number of contigs was higher (Fig. 4). Regardless, the unamplified assembly achieved nearly complete coverage for the genomes of all mock community members.

All programs resulted in fragmented assemblies for the MDA libraries, characterized by one to two magnitude lower maximum contig length and N50 values than the unamplified assembly. Additionally, a relatively small proportion of reads were successfully assembled. However, even with this fragmentation, there were variations. Bulk MDA and emulsion MDA libraries’ assemblies tended to produce higher number of contigs than primase MDA (Fig. 4). In terms of genome coverage, bulk MDA and emulsion MDA assemblies covered up to 50% of the combined mock community genomes, whereas primase MDA showed negligible coverage. Bulk MDA using SPAdes with 40× *k-*mer depth normalization performed best, providing the highest genome coverage between MDA libraries and among assemblers (Fig. 4). Detailed analysis of the bulk MDA assemblies revealed a higher proportion of the genomes covered by contigs with fewer assembly errors for low GC genomes, such as *S. aureus* and *L. monocytogenes* (Supplementary Fig. S8). Specifically, MEGAHIT exhibited the poorest performance, with contigs covering <40% of the reference genomes, nearly two times lower than SPAdes (Supplementary Fig. S8). In contrast, the high GC content mock community member *Pseudomonas aeruginousa* had the lowest proportion of its genome aligned by contigs in all the bulk MDA assemblies (Supplementary Fig. S8). This observation is consistent with the overall underrepresentation of this genome (Fig. 2).

**Figure 5:**
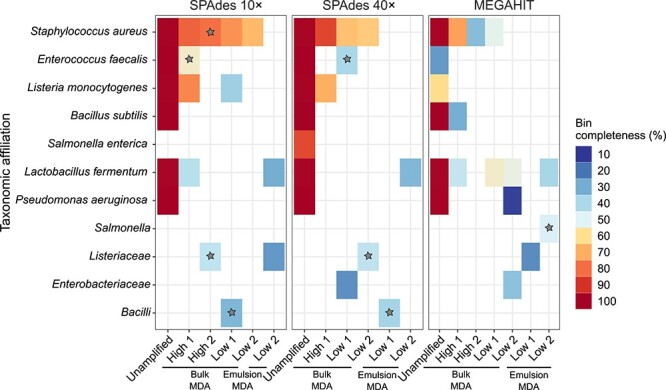
Unsupervised binning results for the unamplified, bulk MDA, and emulsion MDA libraries of the mock community; tiles with gray star correspond to bins having 5%–10% contamination; the remaining tiles correspond to bins with <5% contamination; bin quality was based on completion and contamination as described elsewhere [54]: “high-quality” was defined as >90% completion and <5% contamination, “medium-quality” as ≥50% completion and <10% contamination, and “low-quality” as <50% completion and <10% contamination.

In the unamplified library assemblies, SPAdes showed superior performance by generating high-quality bins for six (SPAdes 10×) to seven (SPAdes 40×) of the eight bacterial mock community members compared to MEGAHIT (Fig. 5). Regarding the MDA libraries, assemblies with SPAdes for the bulk MDA libraries, generated medium-quality bins that exhibited a range of 50%–87% completeness in the high and low DNA-input libraries for *S. aureus.* However, the presence of other mock community members was inconsistent between replicates, and the completeness was often below 50%. Emulsion MDA produced low quality bins (<50% completeness and <10% contamination) for the low input DNA assemblies. Primase MDA did not yield bins despite efforts to recover them using a bin-score equal or above 0.3 in DASTool.

SPAdes was selected to assemble the borehole fluid and groundwater environmental samples. In the case of the borehole fluid, the assembly proceeded without the need of coverage normalization. The proportion of assembled reads in borehole fluid unamplified library's (80%) was 1.6 times higher than in the MDA-amplified libraries (up to 50%; Supplementary Fig. S9A), except for one low DNA input primase MDA library (>70%). However, the high DNA input bulk MDA and emulsion MDA libraries showed assembly metrics similar to the unamplified sample, with maximum contig length of >10^5^ bp and almost the same N50, number of contigs, and predicted genes. These metrics were closely followed by the low DNA input bulk MDA and emulsion MDA assemblies. Primase MDA displayed better assembly statistics for high DNA input libraries compared to lower DNA input. Overall, bulk MDA and emulsion MDA showed better assembly metrics than primase MDA (Supplementary Fig. S9A). Conversely, the groundwater libraries were assembled using 40× coverage normalization, due to the extremely high coverage imbalance that strained the assembler program (Supplementary Fig. S9B). All of the groundwater libraries generated highly fragmented assemblies, with a similar number of assembled reads, smaller maximum contig length (up to 10^4^ bp), and N50 values. However, the total number of contigs was higher in the bulk MDA and emulsion MDA, generating more predicted genes than primase MDA (Supplementary Fig. S9B).

Unsupervised binning of the unamplified borehole fluid library resulted in four high quality and three medium quality bins (Supplementary Fig. S10), including *Desulfosporosinus* and *Desulfovibrio* taxa previously identified in this sample with HMM profiles (Fig. 1B). Other bins from the unamplified sample could not be classified to the genus level. High DNA input libraries of bulk and emulsion MDA produced three medium and two high quality bins affiliated with *Desulfovibrio*, *Deltaproteobacteria* (newly *Desulfobacterota*), and *Bacteriodales* similar to the unamplified assembly. One primase MDA library generated a single low-quality bin with <20% completeness. The missing assemblies indicated libraries were bins that were not generated according to the specific parameters used in DASTool (Supplementary Fig. S10). Unsupervised binning of the groundwater libraries did not result in suitable quality bins.

### Functional profiles

**Figure 6:**
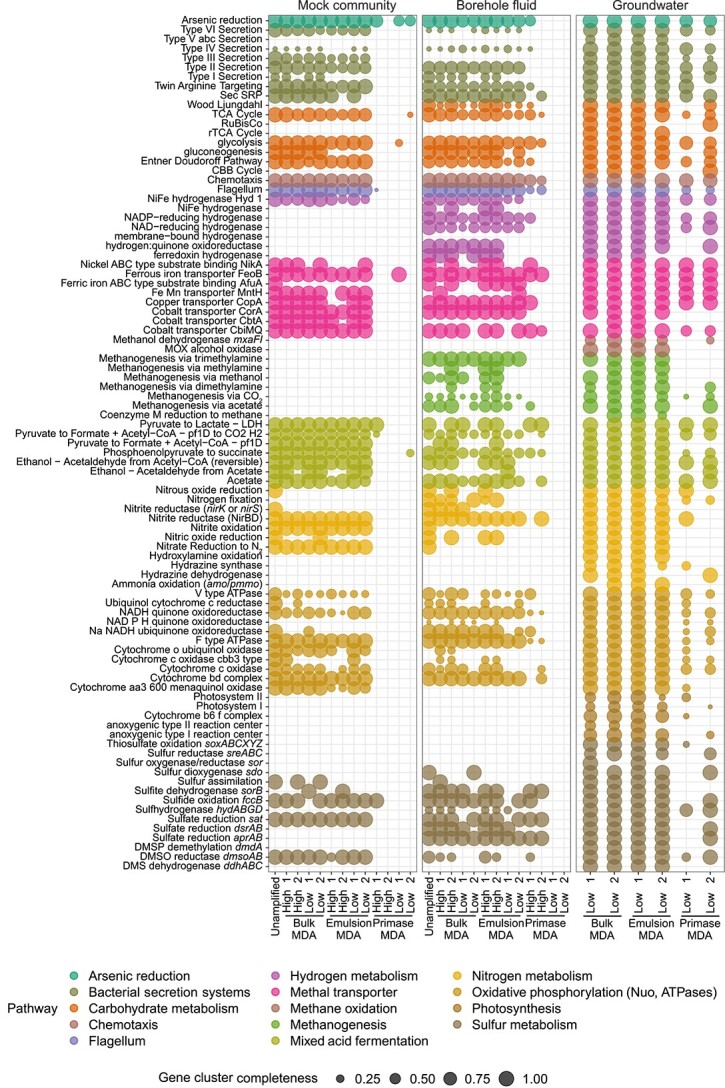
Assembly-based functional profiles showing the presence–absence and completeness of gene clusters for the mock community, borehole fluid, and groundwater libraries; gene clusters involved in the same functional pathway are highlighted using the same color.

The functional profiles were compared in terms of the quantity of annotated genes and the completeness of functional gene clusters to assess the consistency between the MDA amplified assemblies for each sample. In the case of the mock community and borehole fluid, the comparison included an unamplified assembly, serving as a control for the expected outcome. For the mock community, the predicted genes from the SPAdes 40× assemblies were annotated. The mock community unamplified assembly had the most complete number of gene clusters (Fig. 6). Bulk MDA and emulsion MDA assemblies produced similar gene prediction annotations to the unamplified assembly (*p* = .94 and *Pp*= .89, respectively). However, both MDA methods lacked several functional gene clusters, such as those for denitrification processes (nitrite reduction, nitric oxide, and nitrous oxide reduction), which were present only in the *P. aeruginosa* genome within the mock community. Primase MDA assemblies produced the lowest number of gene clusters may be due to the low numbers of contigs and predicted genes (Fig. 5).

The functional profiles from the borehole fluid bulk and emulsion MDA libraries were similar to the unamplified library (*Pp*= .98 and *p* = .59, respectively; Fig. 6). However, several genes were present in the high-input libraries of the bulk MDA and emulsion MDA methods but not in the unamplified library, such as those involved in methanogenesis via methylamines and trimethylamines (*mtbB* and *mtmB*), and *cbb_3_*-type cytochrome c oxidases active in micro-oxic conditions (*ccoPQNO*). Low-input DNA libraries of the primase MDA did not display any function-associated genes, and the high-input DNA libraries had less consistent profiles among replicates (Fig. 6).

For the natural groundwater sample, only low DNA input MDA libraries were feasible due to its very low biomass (Table 1). Both bulk and emulsion MDA libraries showed very similar profiles between replicates and MDA methods, with only few gene clusters missing sporadically across the assemblies. The number of pathways detected in the bulk and emulsion MDA showed no statistical differences between them (*p* = .99; Fig. 6). Compared to mock community and borehole fluid, the groundwater sample had more metabolic functions annotations due to its higher diversity, as read-based taxonomic profiling indicated (Fig. 1C). In contrast, primase MDA assemblies were assigned relatively few pathways (Fig. 6).

Based on the results of the functional profiles, a wide range of metabolic functions were predicted for the MDA-amplified assemblies. Although there was weak statistical evidence for a difference between low and high DNA input (*p* = .05), the small *p* value, combined with the relatively small number of replicates, suggests that DNA input could still account for differences in the functional profiles. In contrast, there was a significant difference between the MDA methods (*p* = <.001). There was no interaction between the MDA method and the DNA input (*p* = .7).

## Discussion

Our study provides insights into the performance and limitations of MDA methods in the context of metagenomic analyses. First, using a defined mock community, we evaluated distinct patterns in read distribution, genome coverage, amplification uniformity, or bias across three different MDA methods (bulk MDA, emulsion MDA and primase MDA) when using two extremely low DNA input levels (~1 pg and 100 pg). The unamplified library served as a baseline, exhibiting expected results of read distributions and providing nearly complete and uniform coverage for the reference genomes in the mock community. In contrast, all the MDA-amplified libraries displayed uneven read distribution and bias against genomes with GC content above 45%. This GC content bias has been reported as a technical limitation during amplification with polymerases [16, 43], which is lower for MDA (3-fold, Phi29 polymerase) over PCR protocols (10^2^- and 10^6^-fold, *Taq* polymerase) [19]. Bulk and emulsion MDA demonstrated higher coverage uniformity for low GC content genomes. However, these genomes exhibited increased variability in the regions overamplified. Naturally occurring read repeats are expected during sequencing, usually increased after amplification, such as in MDA [17, 19]. Overamplification causes some reads to repeat disproportionately, which can affect downstream analyses.

Previous studies suggested that primase and emulsion MDA could improve genome coverages and decrease amplification bias [4, 7, 13, 26, 27]. Some of these studies have used a vortex to create emulsions for the amplification reaction of *E. coli* genomic DNA [26] or single-bacterial cells [58]. Still, our results showed no significant improvement with this protocol compared to bulk MDA. Vortexing is a simple method to create an oil-in-water emulsion after combining the reaction mix with a surfactant that typically generates a broad distribution of droplet size, each of which will work in parallel as separate “microreactors” [59]. For its simplicity and presence in many labs, the use of vortex has been tested to compartmentalize various biological reactions, such as conventional PCR [59-61], single-cell isolation and culturing [62], as well as single-cell whole-genome amplification [58]. Specifically, the emulsion MDA can be prepared with any reaction mix, either primase MDA [29] or standard MDA, and vortexing is a low-cost alternative to microfluidic devices or microdroplet generators. Optimization parameters like the ratio of surfactant to reaction mix, vortexing speed and duration, vortexing technique (pulsing or continuous), shape of the tube, and co-adjuvants like hydrogel particles or beads influence the emulsion quality, ensuring more uniform and smaller droplet size [58, 60, 62, 63]. These parameters must be revisited to demonstrate the advantage of the reduced MDA reactions using a vortex in future studies.

Primase MDA libraries mapped uniquely to selected regions of the *S. aureus* genome at high mapping depth, and minimal overlap between those regions in the replicates. A previous study did not find any difference between bulk MDA and primase MDA in high GC content regions of amplified human cell genomic DNA [27], and another one detected over and underamplification of different regions also in human cell lines [28]. Additional research found that primase MDA induced systematic bias consisting of overamplification of small circular genomes, but no extreme GC content bias [7]. More recently, primase MDA was tested in a microfluidic-based metagenomic assessment by partitioning an environmental sample into subsamples with 5–10 cells and successfully assembling 98 archaeal MAGs. The discrepancy with our results may be due to differences in the nature of the DNA template and DNA starting material, as well as modifications to the method that could be further optimized.

Chimeric sequences occur during the MDA reaction, and the mechanism for chimera formation has been described elsewhere [5, 23, 64]. A small proportion of unmapped reads in our MDA-amplified libraries were identified as chimeras (below 1%). Most detected chimeras were identified as inverted, and the segments were adjacent in the original template. Previous studies using MDA found chimera proportions often exceeded 10% to 50% [6], or chimera rate of 1 in 10 kbp and 1 in 20 kbp [14], and no evidence of chimera formation [17].

DNA contamination is also a known problem during MDA amplification, involving the introduction of exogenous or external DNA from the sample. In our study, <0.6% of the unmapped reads were attributed to taxa unrelated to the mock community members. In contrast, amplification was only observed in the NTCs when using the bulk MDA. The contaminant reads in the NTC mainly contained human DNA or skin-associated bacteria, suggesting contamination during sample handling. Previous studies have addressed endogenous contamination arising from MDA reagents, particularly the Phi29 polymerase [6, 18, 65]. However, no vector or host cell DNA was detected in our study’s NTC libraries, excluding the kit and host cells as sources of contamination.

Endogenous or exogenous contaminants were undetectable in Emulsion and Primase MDA. This distinction may be attributed to different factors in these modified MDA methods. Emulsion MDA minimizes the risk of unintentionally amplifying contaminants by limiting the polymerase's contact area to the few DNA sequences isolated within individual droplets [21]. Primase MDA does not employ synthetic random primers but instead relies on the enzyme primase that detects a specific sequence to initiate priming [3, 27]. This specificity in primase MDA might prevent the initiation of amplification from human DNA or fragmented DNA sequences. On the contrary, bulk MDA, where the polymerase is dispersed throughout the reaction mix, there is a higher likelihood of contact with exogenous DNA, such as human DNA or cross-contamination from other samples.

Assuming all the unmapped reads consist of exogenous contaminants, including those unclassified, it represented a small proportion of the total library reads. These results alongside the low proportion of detected contaminant in the NTCs of bulk MDA, as well as the absence of amplification in both bulk MDA and primase MDA, show that the contamination in the amplified libraries did not significantly influence or skew the taxonomic profile. Instead, it highlights the remarkable strength of Phi29 polymerase to amplify extremely low-DNA samples and how the modified MDA methods might resolve the contamination problems.

Others have reported the advantage of decontaminating the MDA reagents, materials, and working area with UV irradiation in eliminating the amplification of contaminating DNA, improving whole genome amplification from single cells or low microbial biomass samples [66, 67]. Our study implemented a thorough decontamination protocol for surfaces, PCR water, and materials used for MDA reactions involving sodium hypochlorite and 80% ethanol, followed by UV radiation. These measures could also contribute to the low levels of contaminants detected in the MDA-amplified libraries mock community.

Other nonspecific amplifications include spurious sequences, hereafter referred to as artifacts. The artifacts introduced during amplification protocols involving synthetic primers (such as PCR and standard MDA) are attributed to nonspecific DNA synthesis following primer dimer formation [5, 15, 16, 18] and stochastic effects [9, 14, 68]. Between 3% and 7% of the MDA-amplified libraries of the mock community could not be classified. The unamplified sample had an even lower proportion of unmapped reads, <2% of which were not classified. Most unidentified unmapped reads in the MDA-amplified libraries could represent artifacts introduced during amplification.

Nonspecific amplifications like chimeras, artifacts, and contaminating DNA similar to the GC and amplification bias also affect downstream analysis, mainly in de novo applications, by corrupting the library during the reconstruction of contigs and bins, leading to misrepresentations of the community. Our downstream analyses were conducted on reads that underwent rigorous quality checks within the ATLAs metagenome pipeline containing diverse error correction tools [35]. This critical step may significantly reduce spurious sequences and, consequently, the low proportion of these nonspecific amplicons.

Several assemblers, including MEGAHIT and SPAdes, also integrate error correction strategies like k-mer counting to filter out nonspecific, low-depth kmers [45, 46, 69]. However, coverage imbalance due to amplification bias can be another challenge during the assembly. We compared MEGAHIT and SPAdes assembly metrics in our mock community libraries to determine the optimal assembler for handling these specific challenges in the MDA-amplified environmental samples. We chose both assemblers based on their recognized use in simple to more complex environmental metagenome studies [70]. To mitigate the coverage imbalance and prevent the accidental removal of true low-depth reads, we adopted a strategy of coverage normalization to 10× and 40× before SPAdes assembly. This optional parameter in the ATLAS pipeline also helped optimize computational resources. Our comparative analysis found that SPAdes was the preferred assembler for the environmental samples due to its superior performance. Normalization at 40× was used whenever required to improve assembly.

Mock community and environmental samples were employed to compare the results of read-based taxonomic profiles using two single-copy genes and an assembly-based approach to evaluate functional profiles and binning. The unamplified in the mock community and borehole fluid libraries served as a comparative control to their same samples’ MDA-amplified libraries.

MDA-amplified libraries within all samples displayed differential representations of taxa. Increased misrepresentation and abundance skewing were observed at low-DNA input and when using primase MDA, underscoring the importance of both the MDA method and DNA input when assessing the impact on taxonomic representation. Previous research found MDA-mediated representational bias in three environmental samples through 16S rRNA gene-amplicon sequencing, using higher DNA starting amounts (>1 ng) than our study [2]. Another study also reported bias higher in standard MDA over primase MDA when comparing 16S rRNA gene profiles in environmental samples and 16S rRNA gene fragment DGGE profiles in a defined mixture of six species [15]. Additional research also found misrepresentation bias in a mock assemblage of seven DNA viruses using qPCR and human saliva DNA viromes using high-throughput sequencing; however, dominant taxa closely resembled those from unamplified samples [7]. Our study revealed primase MDA exhibited the least favorable performance. This outcome is consistent with prior research on human cell lines reporting significant representation bias in specific regions when amplifying using the same primase MDA kit used in our study [28].

Although bulk MDA and emulsion MDA lead to relative abundance skewing, the capacity to detect comparable taxon, borehole fluid at the genus level, and groundwater at the class level suggests that the bias might not have been severe enough to completely obscure the diversity at least at higher taxonomic resolution. These is important to potentially understand the community composition in an environmental sample when using extremely low DNA starting material. A previous study also found a relatively uniform detection of major phyla by 16S rRNA gene clone libraries in MDA-amplified contaminated soil samples that had extremely initial low DNA-density [17].

SPAdes was the preferred assembler because it achieved better assembly metrics and bin quality after comparing the MDA-amplified and unamplified mock community libraries. Unlike the expected unamplified libraries that demonstrated superior metrics, the MDA-amplified libraries consistently produced fragmented assemblies, highlighting the impact of the MDA bias and nonspecific amplifications on assembly quality. However, bulk MDA and emulsion MDA generally exceeded the performance of primase MDA. The assemblies from bulk MDA at high DNA input for the mock community and borehole fluid libraries yielded medium- to low-quality bins, indicating the potential for using MDA in simple microbial diversity to access some metagenome-assembled genomes. However, these preliminary results could benefit from the utilization of more up-to-date bioinformatics tools to obtain more bins or improve the accuracy, completeness, and robustness of the obtained ones.

Mock community and groundwater MDA-amplified libraries required coverage normalization, but borehole fluid did not, implying less coverage imbalance. Less complex communities like the borehole fluid, with fewer highly abundant taxa, could exhibit less bias due to a more evenly amplification of their genomic DNA. However, the mock community, a simple community like the borehole fluid, required a coverage normalization before assembly. Variations in DNA abundance across diverse taxa, disparities in GC content, and differences in genomic features within microbial communities could also influence the amplification with MDA leading to different coverage imbalance between samples. In contrast, MDA amplification, especially bulk and emulsion methods, performed relatively better in maintaining functional gene annotations similar to unamplified assemblies for high-DNA input (around 100 pg) and in low DNA starting material, as observed in the groundwater. These show the promising use of the MDA-amplified samples for functional profiling, giving insights into metabolic pathways and gene functions. However, our interpretation must be cautiously approached, mainly due to the limitations of low replication. This constraint is evident in the inconsistent performance, evidenced by variability and a lack of specific functional gene clusters between replicates. Such inconsistencies underscore the need for more extensive replication to validate our findings.

This study represents a proof of concept, demonstrating the potential of bulk MDA for amplifying low DNA environmental samples for metagenomic analysis. It demonstrates the ability to gain insights into these samples’ taxonomic and functional composition. It balances assembly efficiency and genome coverage, making it a valuable tool for studying microbial communities with simple to moderate complexity in conventional laboratories. Optimizing MDA methods may be necessary for more complex microbial communities, such as reducing the reaction volume or further refining the emulsion MDA technique to take advantage of the compartmentalization of the reaction, applicable to both conventional or primase MDA methods. Assessing the complexity of communities in environmental samples using 16S rRNA gene amplicon sequencing before and after MDA could also be recommended to identify whether amplified nucleic acids adequately represent the original community before high-throughput sequencing. This alternative could help with meaningful interpretations, protocol optimization, gauge amplification bias, and improve metagenome downstream tools and analysis.

The use of MDA-amplified samples could benefit a range of genomic analyses, including hybridization capture techniques. These methods could use the increased quantity of MDA-amplified DNA fragments, which usually are long, thus preserving the essential structural information of the genome, particularly advantageous for techniques that require high-quality genomic material. Hybridization capture, also known as targeted sequence capture, is an enrichment method employing biotinylated DNA or RNA baits that bind to specific DNA sequences of interest [71]. This technique helps isolate and sequence genomic regions from DNA libraries [72] and has been used to enriched 16S rRNA genes [71, 72], metabolic genes [73], and entire genomes from metagenomic samples [74]. This approach reduces DNA extraction, PCR amplification, and sequencing biases [71]. Future research should investigate if preamplifying samples with MDA before hybridization capture can help minimize MDA-related biases, thereby enhancing phylogenetic resolution and aiding the discovery of novel prokaryotic taxa in low biomass samples.

Modifications and /or adaptations of the MDA technique are being designed and released such as ResolveDNA Microbiome (Alpha) - BioSkryb Genomics and “Primary Template-directed Amplification” method [75], which promising applicability need evaluation to demonstrate their efficiency in amplifying DNA from various environmental samples, especially those with low DNA content. Researchers must be mindful of biases and limitations, especially concerning GC content and microbial community complexity, and use MDA as an alternative for WGA when the DNA concentration prevents direct sequencing. Also, depending on the intended scope of the study, studies should carefully weigh whether to use MDA, mostly in cases where quantifiable parameters from metagenomic data must be evaluated.

Overall, our study demonstrated how different MDA protocols impact metagenomes from samples with extremely low DNA content and influence taxonomic and functional profiles of samples of varying compositional diversity. Moreover, our study has identified potential biases likely to affect metagenomes from amplified DNA from environmental samples. With caution, we suggest that bulk MDA can be used to obtain environmental sample metagenomes when limited DNA concentrations preclude direct sequencing. This research provides a framework for advancing microbial community research of low biomass or challenging-to-access habitats.

## Supplementary Material

Ospinoms_SI_ycae024

## Data Availability

All sequence data were deposited in Genbank under BioProject accession ID PRJNA695014.
