# Supplementary material for: Evaluation of multiple displacement amplification for metagenomic analysis of low biomass samples
Source: ISME Commun. 2024 Feb 12;4(1):ycae024. doi: 10.1093/ismeco/ycae024 (PMC10945365; doi:10.1093/ismeco/ycae024)
Supplement: Ospinoms_SI_ycae024 [file ospinoms_si_ycae024.docx]

**Evaluation of multiple displacement amplification for metagenomic analysis of low biomass samples**

Melody Cabrera Ospino^1^, Katja Engel^1^, Santiago Ruiz Navas^1^, W. Jeffrey Binns^2^, Andrew C. Doxey^1^, Josh D. Neufeld^1^*

^1^Department of Biology, University of Waterloo, Waterloo, Ontario, Canada

^2^Nuclear Waste Management Organization of Canada, Canada

*Corresponding author: Department of Biology, University of Waterloo, 200 University Avenue West, Waterloo, Ontario, N2L 3G1, Canada. Tel.+1 519-888-4567; Fax +1 519-746-0614

E-mail: [jneufeld@uwaterloo.ca](mailto:jneufeld@uwaterloo.ca)

**Supplementary Data 1.** Parameter options executed in the ATLAS pipeline for quality checking, trimming, assembly, and binning.

########################

# Quality control

########################

**data_type**: metagenome # metagenome or metatranscriptome

# remove (PCR)-duplicated reads using clumpify

**deduplicate**: true

**duplicates_only_optical**: false

**duplicates_allow_substitutions**: 2

# used to trim adapters from reads and read ends

**preprocess_adapters**: /Data/reference_databases/atlas/2.1.x/adapters.fa

**preprocess_minimum_base_quality**: 10

**preprocess_minimum_passing_read_length**: 51

# 0.05 requires at least 5 percent of each nucleotide per sequence

**preprocess_minimum_base_frequency**: 0.05

**preprocess_adapter_min_k**: 8

**preprocess_allowable_kmer_mismatches**: 1

**preprocess_reference_kmer_match_length**: 27

# error correction where PE reads overlap

**error_correction_overlapping_pairs**: true

#contamination references can be added such that -- key: /path/to/fasta

**contaminant_references**:

**PhiX**: /Data/reference_databases/atlas/2.1.x/phiX174_virus.fa

**contaminant_max_indel**: 20

**contaminant_min_ratio**: 0.65

**contaminant_kmer_length**: 13

**contaminant_minimum_hits**: 1

**contaminant_ambiguous**: best

########################

# Pre-assembly-processing

########################

**normalize_reads_before_assembly**: true

# target kmer depth

**normalization_target_depth**: 10 #40 was used as well during the assembly of the mock community with SPAdes

**normalization_kmer_length**: 21

**normalization_minimum_kmers**: 2

**error_correction_before_assembly**: true

# join R1 and R2 at overlap; unjoined reads are still utilized

**merge_pairs_before_assembly**: true

# extend reads while merging to this many nucleotides

**merging_extend2**: 40

# Iterations are performed until extend2 x iterations

**merging_flags**: ecct iterations=5

**merging_k**: 62

########################

# Assembly

########################

# megahit OR spades

**assembler**: spades # for the mock community it was also employed Megahit for assembly.

# Megahit

#-----------

# 2 is for metagenomes, 3 for genomes with 30x coverage

**megahit_min_count**: 2

**megahit_k_min**: 21

**megahit_k_max**: 121

**megahit_k_step**: 20

**megahit_merge_level**: 20,0.98

**megahit_prune_level**: 2

**megahit_low_local_ratio**: 0.2

# ['default','meta-large','meta-sensitive']

**megahit_preset**: default

# Spades

#------------

**spades_skip_BayesHammer**: true

**spades_use_scaffolds**: true # otherwise use contigs

#Comma-separated list of k-mer sizes to be used (all values must be odd, less than 128 and listed in ascending order).

**spades_k**: auto

**spades_preset**: meta # meta, ,normal, rna single end libraries doesn't work for metaspades

**spades_extra**: ''

# Filtering

#------------

**prefilter_minimum_contig_length**: 200

# filter out assembled noise

# this is more important for assemblys from megahit

**filter_contigs**: true

# trim contig tips

**contig_trim_bp**: 0

# require contigs to have read support

**minimum_average_coverage**: 1

**minimum_percent_covered_bases**: 20

**minimum_mapped_reads**: 0

# after filtering

**minimum_contig_length**: 300

########################

# Quantification

########################

# Mapping reads to contigs

#--------------------------

**contig_min_id**: 0.95

**contig_map_paired_only**: true

**contig_max_distance_between_pairs**: 1000

**maximum_counted_map_sites**: 10

########################

# Binning

########################

**final_binner**: DASTool # [DASTool or one of the binner, e.g. maxbin]

**binner**: # If DASTool is used as final_binner, use predictions of this binners

- metabat

- concoct

- maxbin

**metabat**:

**sensitivity**: sensitive

**min_contig_length**: 1500 # metabat needs >1500

**concoct**:

**Nexpected_clusters**: 200 # important parameter

**read_length**: 100 # change this parameter !

**Niterations**: 500

**min_contig_length**: 1000

**maxbin**:

**max_iteration**: 50

**prob_threshold**: 0.9

**min_contig_length**: 1000

**DASTool**:

**search_engine**: diamond

**score_threshold**: 0.3 #Score threshold until selection algorithm will keep selecting bins [0..1].

**SubsetSize**: 500000

**Supplementary Data 2.** Detailed explanation of Kaiju, htSeqTool and custom chimera detection script.

Chimera detection: Using unmapped reads from mock community metagenomes, inverted and direct chimeric reads were identified by using the Burrows-Wheeler Aligner to compare against reference genomes using the mem algorithm with default parameters [36]. The resulting BAM file alignment was used to filter soft-clipped alignments, with a SAM CIGAR value of xxSxxM or xxMxxS with SAM prefix of "SA:Z" [37]. These filtered reads were then used to reconstruct new FASTQ paired-end read files by splitting the reads into two segments following the soft-clipped location. These new files were backtrack aligned to the combined reference genomes using bwa-pe [38]. Chimeric reads were removed with segments (“sub-reads”) of insert size <20 nucleotides, or with one of the segments unambiguously aligned to two or more different genomic regions. The SAM flag values were used to separate inverse and direct sequence chimeras. Segments mapping to different strands of the same reference genome were identified as inverted chimeras and those aligned to the same strand were designated as direct sequence chimeras. The SAM TLEN values were used to determine insert size or distance of the genome spanned by each chimeric segment.

Kaiju program: Fast taxonomic classifier used for the unmapped reads. This tool uses k-mers to identify the taxonomy of the reads by aligning them to the NCBI reference genome database using the default’s parameters. The default e-value cut-off for taxonomic assignment is 0.01.

MDS for coverage profile: The htSeqTools R package measures similarity between the read coverage of samples i and j. The distance calculation process can be summarized in three steps: log transformation, correlation, and distance calculation. The log transformation package transforms read coverage of samples i and j by calculating their log(coverage +1). The log(coverage +1) of samples i and j were used to calculate Pearson correlations (ρij). Distances between samples i and j were calculated as dij = 0.5(1 − ρij). Finally, multi-dimensional scaling (MDS) was used to plot the coverage profiles of each library in two dimensions.

**
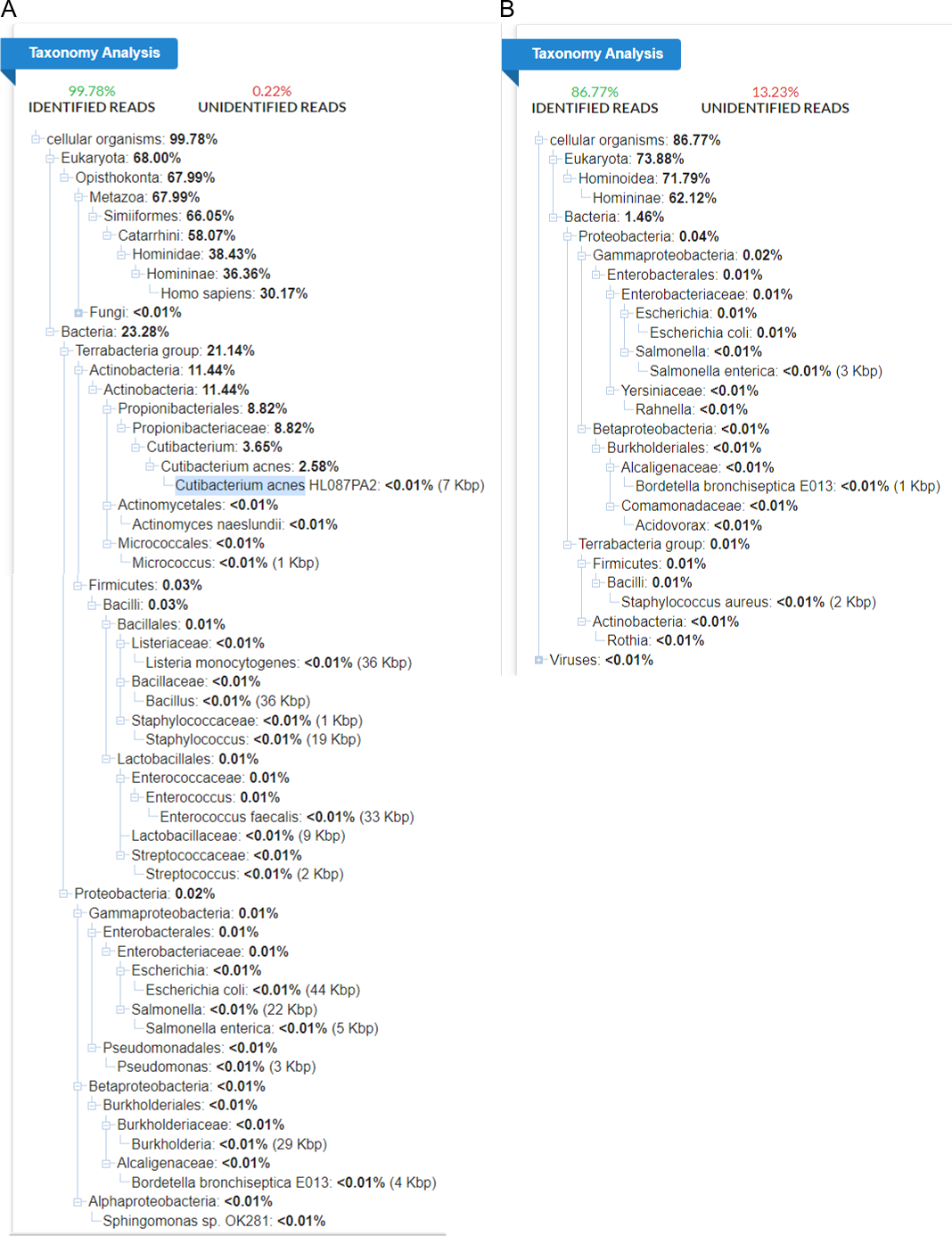
**

**Figure S1.** Taxonomic classification of the raw reads of the No-template controls amplified with bulk MDA using the online Taxonomy Analysis Tool (STAT) from the NCBI SRA. The result are reported as taxonomic hierarchy with the relative abundance composition proportional to the sequence abundance of the genome. (A.) PCR Tris buffer and (B.) PCR water. Image source: The image depicts a screenshot showing the taxonomic analysis results obtained for run SRR13554769 (NTC tris buffer) and SRR13554771 (NTC PCR water) from the Sequence Read Archive (SRA) of NCBI.

**Figure S2.** Principal coordinates analyses of Euclidean distances derived based on Bray Curtis metrics from the mock community (A.), borehole fluid (B.), and groundwater (C.) libraries amplified with bulk MDA, emulsion MDA, and primase MDA, with two DNA input (High and Low), and unamplified. Samples that are closer together have communities that are likely more similar to one another.

**
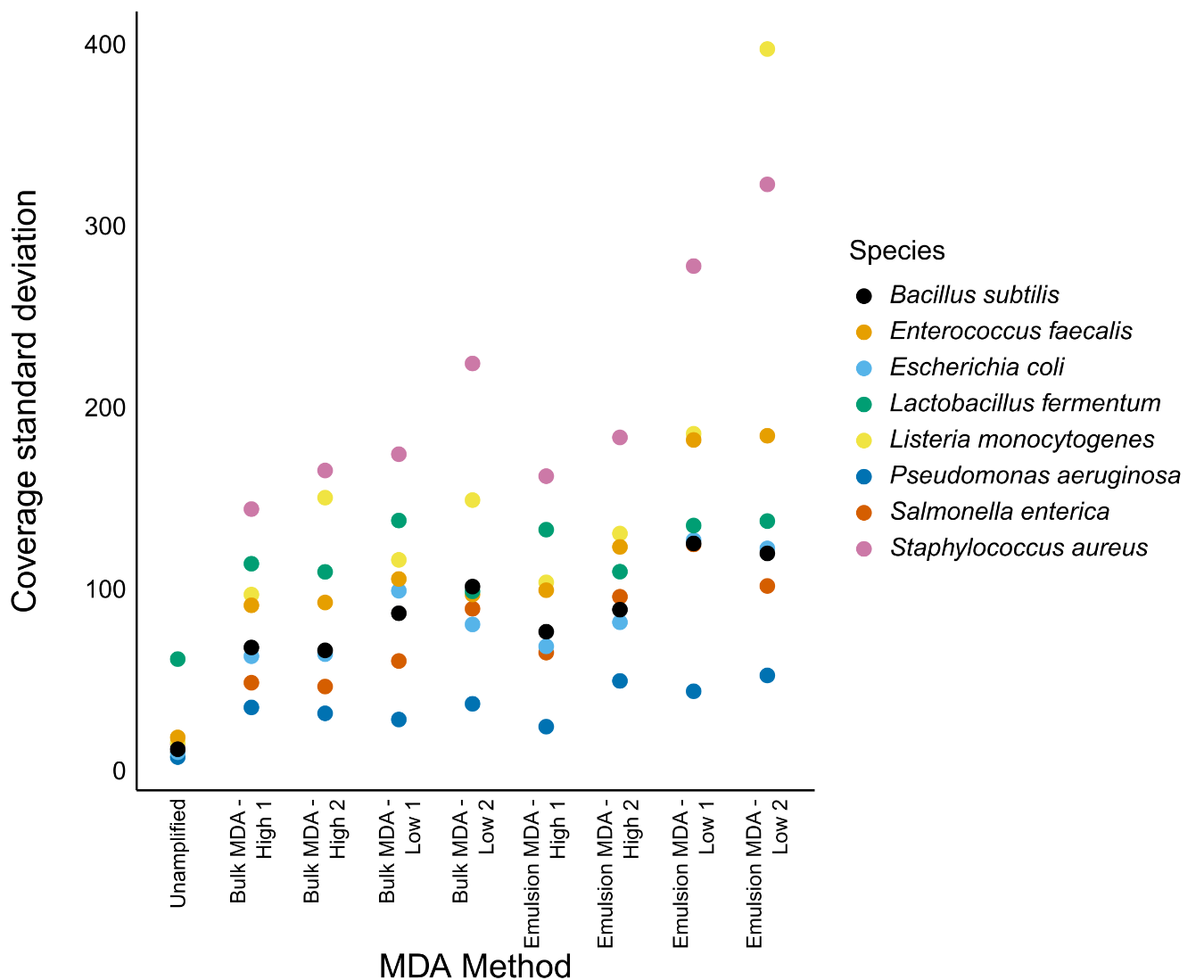
**

**Figure S3.** Coverage standard deviation (ssdcoverage) for the reads mapped to each reference genome of the unamplified and MDA–amplified mock community libraries. The calculation was only possible for bulk and emulsion MDA, since primase MDA lacked reads mapped to most of the reference genomes. Ssdcoverage closer to 0 indicate uniformity distribution of reads along the genome. On the contrary, higher ssdcoverage, imply higher dispersion in the coverage possibly caused by over-amplification.

**Figure S4.** Coverage plots showing the number of reads obtained for each base of the *Staphylococcus aureus* genome in mock community libraries. Panels show coverage profiles for each MDA amplification method overlaid with the unamplified library.

**
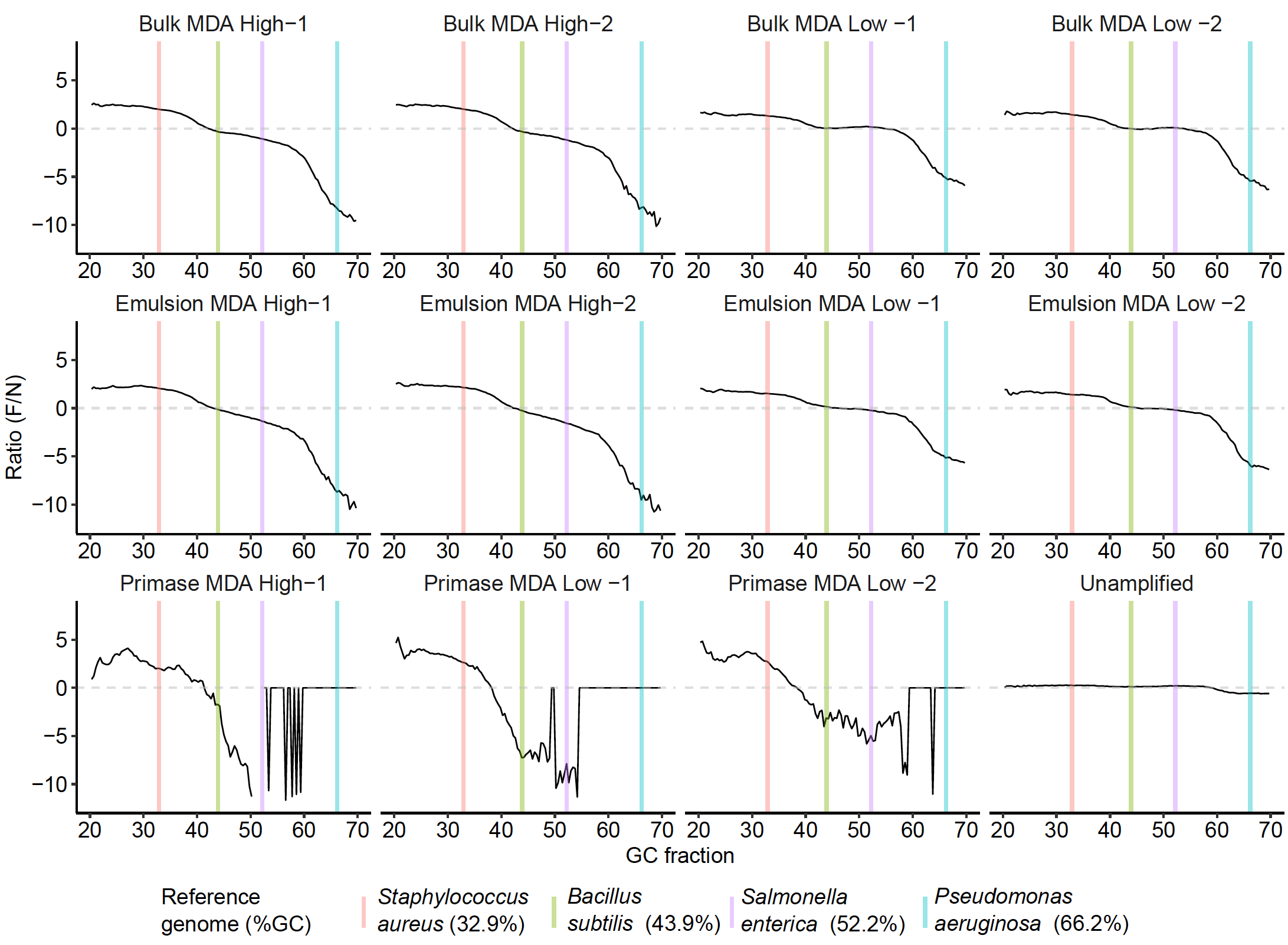
**

**Figure S5.** GC content assessment for the unamplified and MDA-amplified libraries of a mock community sample with different DNA concentration. Each graph shows the normalized (log 2 transformation) observed reads (F) divided by the expected read count (N) of a particular GC fraction. The vertical colored lines mark the average GC content of the four reference genomes assessed in this analysis. Without bias, the observed GC profiles would match the expected profile (i.e., dashed gray line).

**
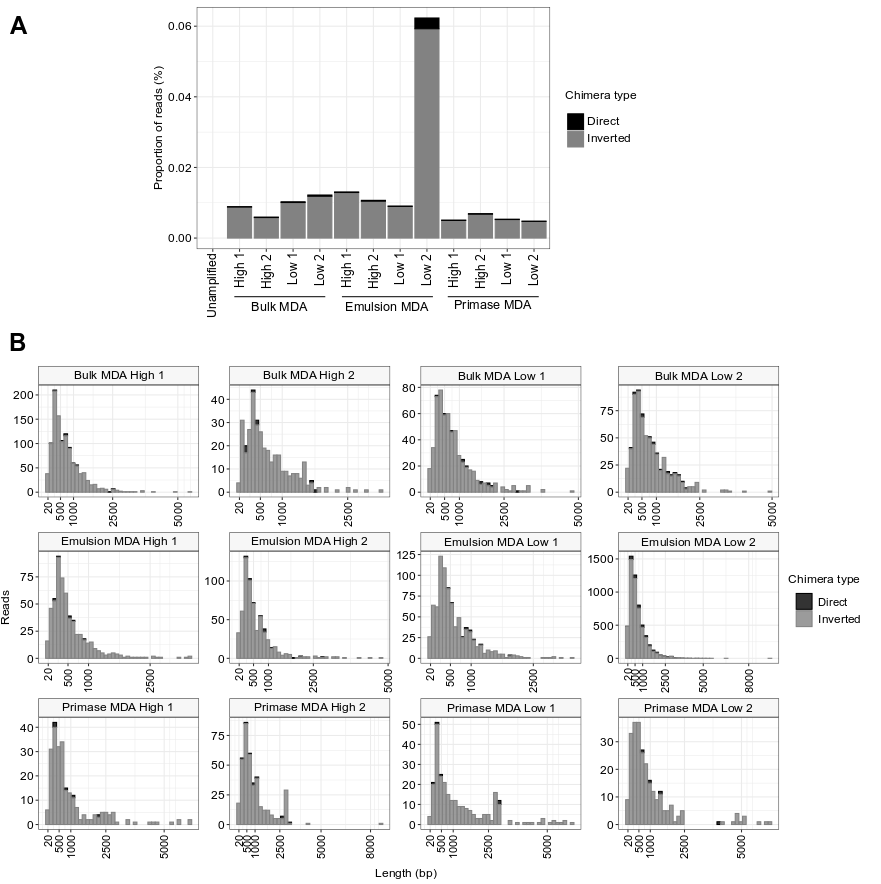
**

**Figure S6.** Proportion of direct and inverted chimeric reads detected in the libraries from the low and high-DNA input mock community samples amplified with bulk MDA, emulsion MDA, and primase MDA (A). The length distribution of the chimeric reads is shown for each library (B).

**Figure S7.** Taxonomic classification for the unmapped reads for each mock community library for the low and high-DNA input amplified with bulk MDA, emulsion MDA and primase MDA. The taxa are assigned to each read using the NCBI taxonomy and a reference database of protein sequences from microbial genomes using the kaiju program. Light gray shaded bars represent genera that were not present in the initial mock community sample. Dark gray shaded bars represent the proportion of unclassified reads.


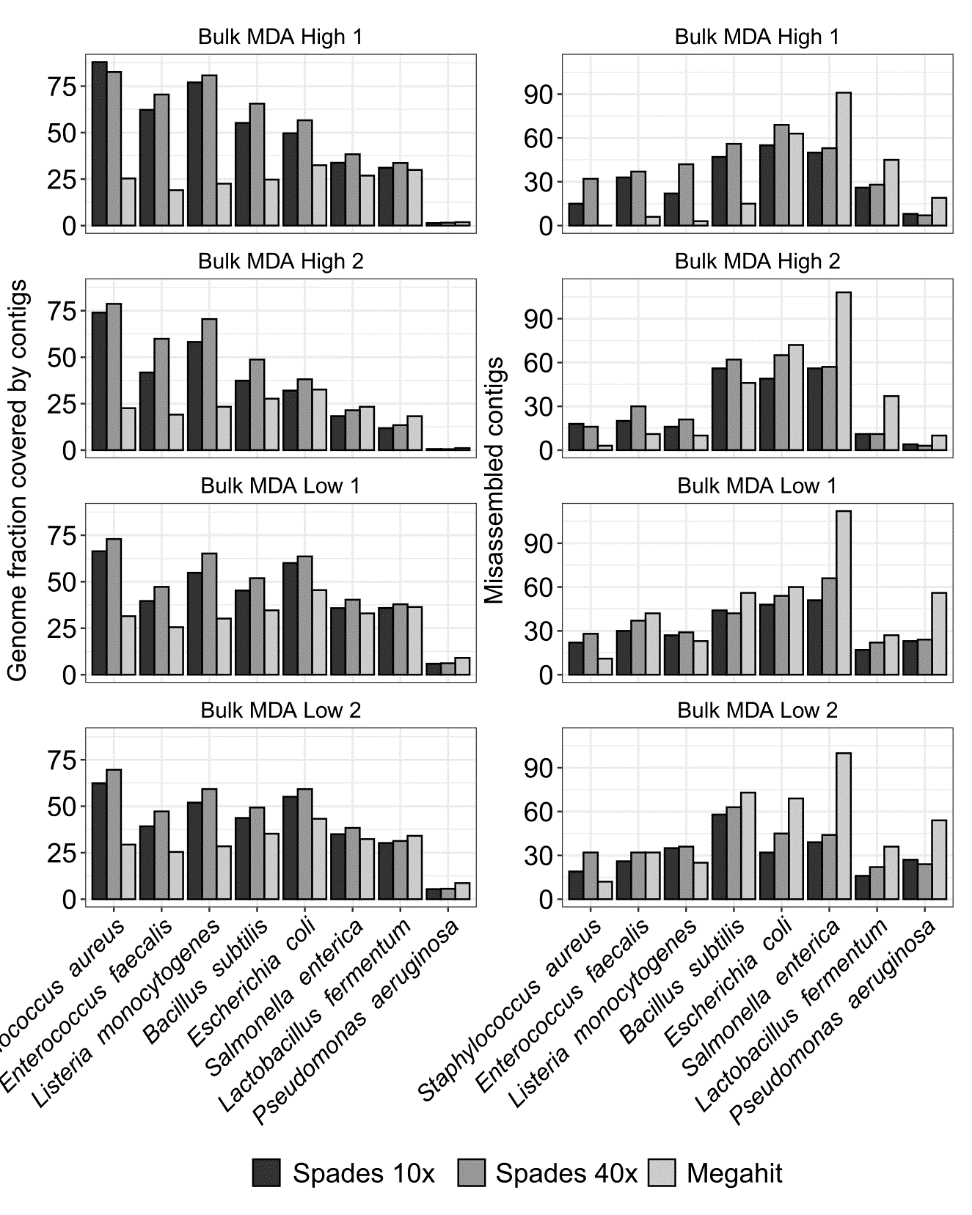


**Figure S8.** Proportions of contigs covering the bacterial reference genomes (left) and misassembled contigs (right). Sequencing libraries were generated using the bulk MDA method with high and low DNA input amounts. Assemblies were performed with SPAdes using 10× or 40× kmers coverage normalization and MEGAHIT. Bacterial genomes are sorted by lowest to highest GC content from left to right.


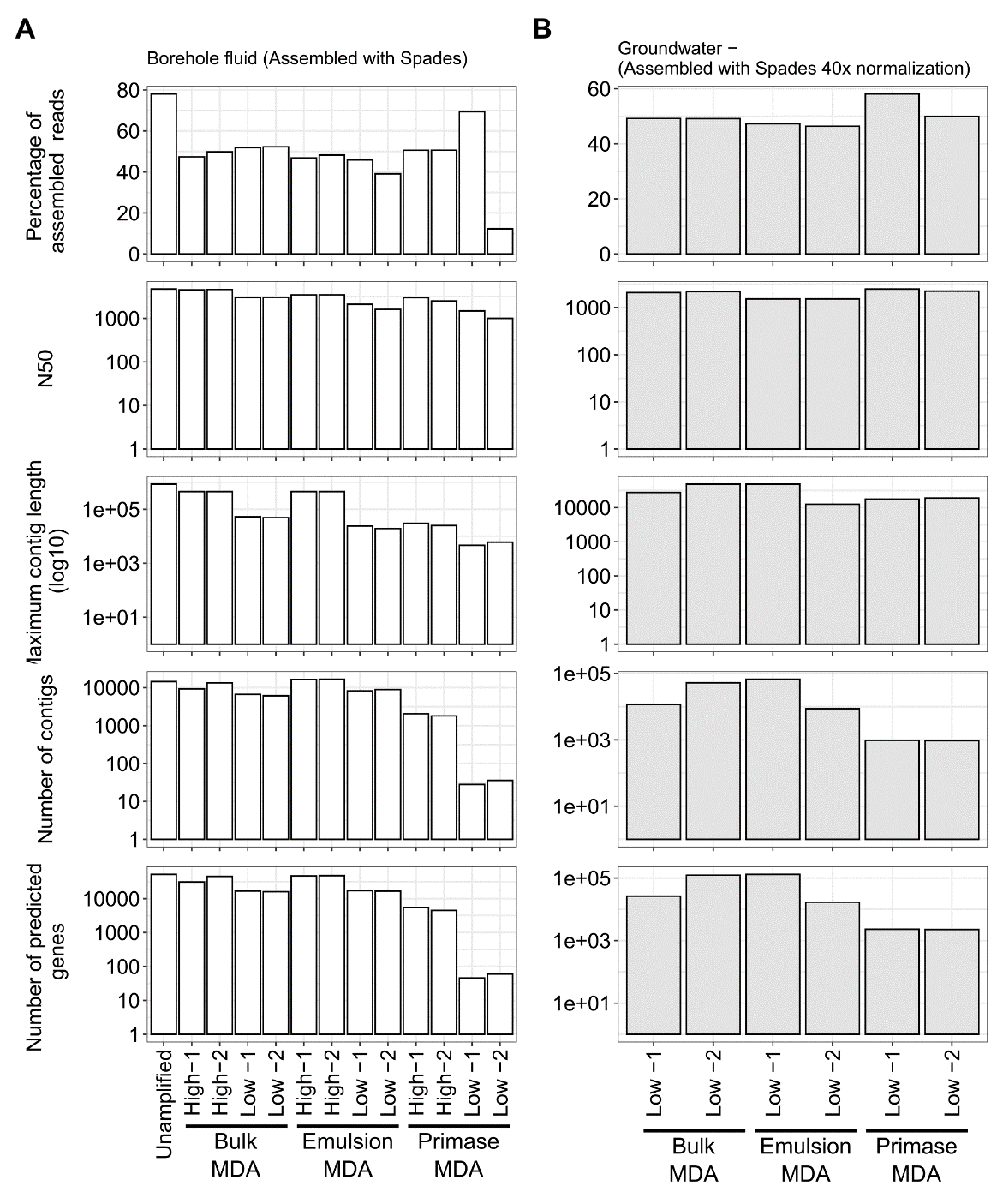


**Figure S9.** Assembly statistics for unamplified and MDA libraries from the borehole fluid (**A**) and groundwater (**B**). High and low-DNA input samples were amplified with bulk MDA, emulsion MDA, and primase MDA in duplicate. The libraries were assembled with SPAdes without normalizing the kmer coverage for borehole fluid (**A**) and with 40× normalization in groundwater (**B**).


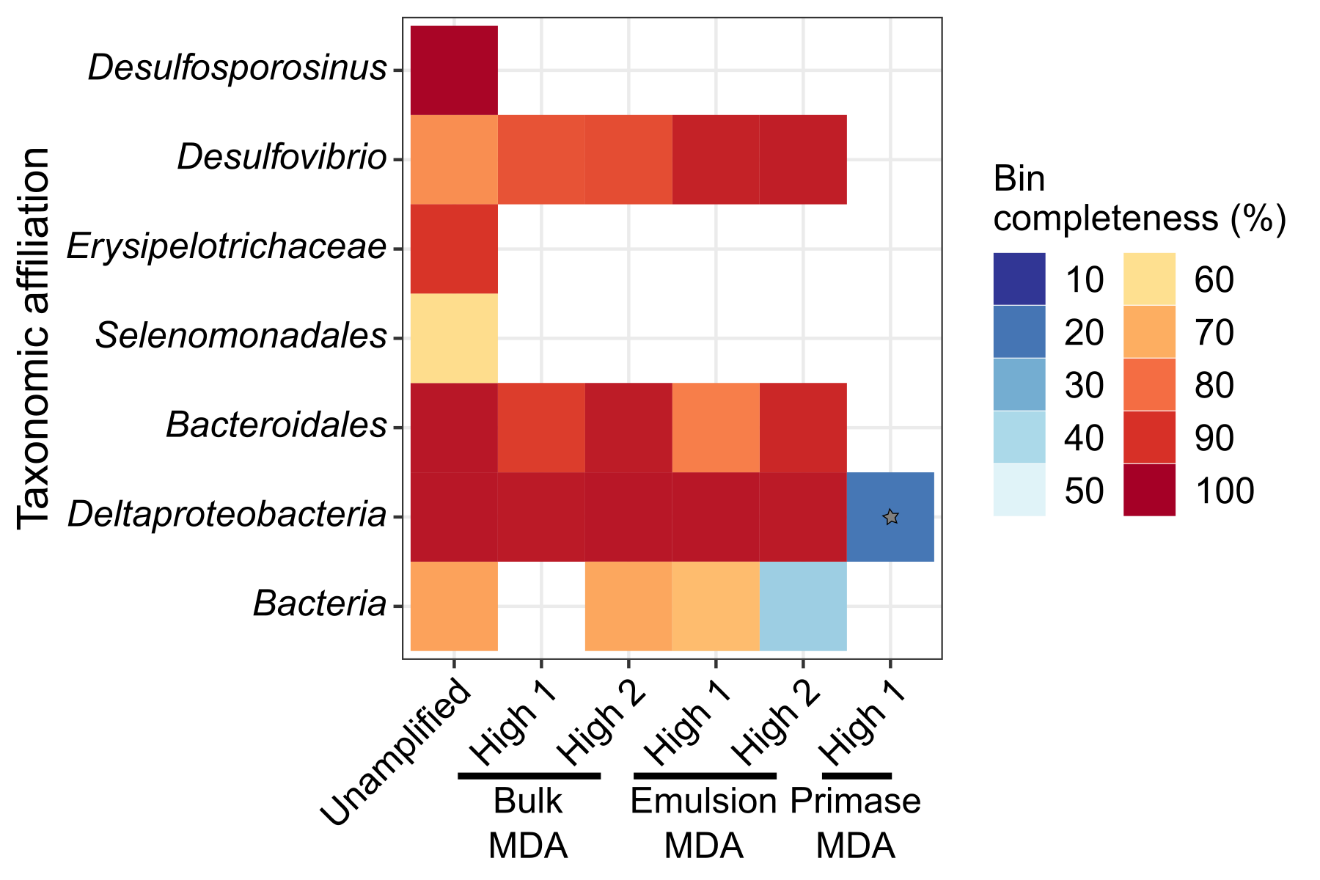


**Figure S10.** Unsupervised binning results for unamplified, bulk MDA, emulsion MDA, and primase MDA libraries of the borehole fluid sample. Only bins with <10% contamination are shown. Tile with gray star correspond to bins having 5-10% contamination. The remaining tiles correspond to bins with <5% contamination.

**Supplementary Table 1.** Library data size in Gb of each sample before and after quality checking and trimming..

|  | Mock community | | | | Borehole fluid | | | | Groundwater | | Control |  |  |  |
| --- | --- | --- | --- | --- | --- | --- | --- | --- | --- | --- | --- | --- | --- | --- |
|  | High | | Low | | High | | Low | | Low | | pcr water | | Tris buffer | |
|  | Raw | QC | Raw | QC | Raw | QC | Raw | QC | Raw | QC | Raw | QC | Raw | QC |
| Bulk MDA | 3.2 | 1.4 | 1.5 | 1.4 | 0.8 | 0.7 | 1.4 | 1.3 | 10.5 | 10.2 | N/A | N/A | N/A | N/A |
|  | 3.1 | 1.5 | 1.4 | 1.4 | 1.4 | 1.4 | 0.9 | 0.9 | 8.9 | 8.7 | N/A | N/A | N/A | N/A |
| Emulsion MDA | 1.5 | 1.3 | 2.3 | 1.3 | 1.3 | 1.3 | 2.3 | 1.3 | 11.5 | 10.8 | N/A | N/A | N/A | N/A |
|  | 1.6 | 1.1 | 2.2 | 1.4 | 1.2 | 1.3 | 2.4 | 1.8 | 10.7 | 10.1 | N/A | N/A | N/A | N/A |
| Primase MDA | 1.1 | 1.2 | 2.4 | 1.3 | 1.3 | 1.1 | 1.4 | 1.4 | 12.0 | 9.8 | N/A | N/A | N/A | N/A |
|  | 1.5 | 1.4 | 2.3 | 1.2 | 1.3 | 1.1 | 1.8 | 1.5 | 10.6 | 9 | 1 | 0.8 | 1.5 | 1.3 |
| Unamplified | 2.0 | 1.8 |  |  |  | 1.6 |  |  |  |  |  |  |  |  |
